# If amyloid drives Alzheimer disease, why have anti-amyloid therapies not yet slowed cognitive decline?

**DOI:** 10.1371/journal.pbio.3001694

**Published:** 2022-07-21

**Authors:** Christian Haass, Dennis Selkoe

**Affiliations:** 1 German Center for Neurodegenerative Diseases (DZNE), Munich, Germany; 2 Metabolic Biochemistry, Biomedical Center (BMC), Faculty of Medicine, Ludwig-Maximilians University, Munich, Germany; 3 Munich Cluster for Systems Neurology (SyNergy), Munich, Germany; 4 Ann Romney Center for Neurologic Diseases, Department of Neurology, Brigham and Women’s Hospital, Harvard Medical School, Boston, Massachusetts, United States of America

## Abstract

Strong genetic evidence supports an imbalance between production and clearance of amyloid β-protein (Aβ) in people with Alzheimer disease (AD). Microglia that are potentially involved in alternative mechanisms are actually integral to the amyloid cascade. Fluid biomarkers and brain imaging place accumulation of Aβ at the beginning of molecular and clinical changes in the disease. So why have clinical trials of anti-amyloid therapies not provided clear-cut benefits to patients with AD? Can anti-amyloid therapies robustly decrease Aβ in the human brain, and if so, could this lowering be too little, too late? These central questions in research on AD are being urgently addressed.

## Abstract 2

Evidence suggests that an imbalance between production and clearance of amyloid-beta is an early, invariant feature of Alzheimer disease that drives its neuronal and glial pathology and precedes cognitive symptoms. So why are we still unable to slow cognitive decline with anti-amyloid therapies?

## Introduction

What can be worse than “losing oneself”? These were the famous words that Auguste Deter, the first known patient with the type of dementia now called Alzheimer disease (AD), used to describe her mental deficits when she met Alois Alzheimer around 1901 [1]. At that time, she was the first patient documented with this progressive form of dementia; now, we expect to have well over 50 million patients with AD by the year 2050 [2] if no preventive treatments are found soon. AD is a major threat to our aging society and will be even more so in the future as life expectancy rises. Scientists from many different disciplines have worked intensively over 4 decades to try to identify the triggers of the disease and, based on these findings, to develop therapeutic strategies. However, although many clinical trials using approaches based on seemingly well-identified targets have been conducted [3], none of them seems to have reached its final goal: to substantially slow cognitive decline. This dispiriting news has led some to conclude that decades of intense research have failed because scientists wasted their time focusing on the wrong mechanism. But is this really true? Do we indeed have no idea what triggers AD? Were all clinical trials a failure? In other words, did we simply lose valuable time by working on the wrong targets, and are there mysterious “alternative pathways” that scientists have entirely missed so far?

**Fig 1:**
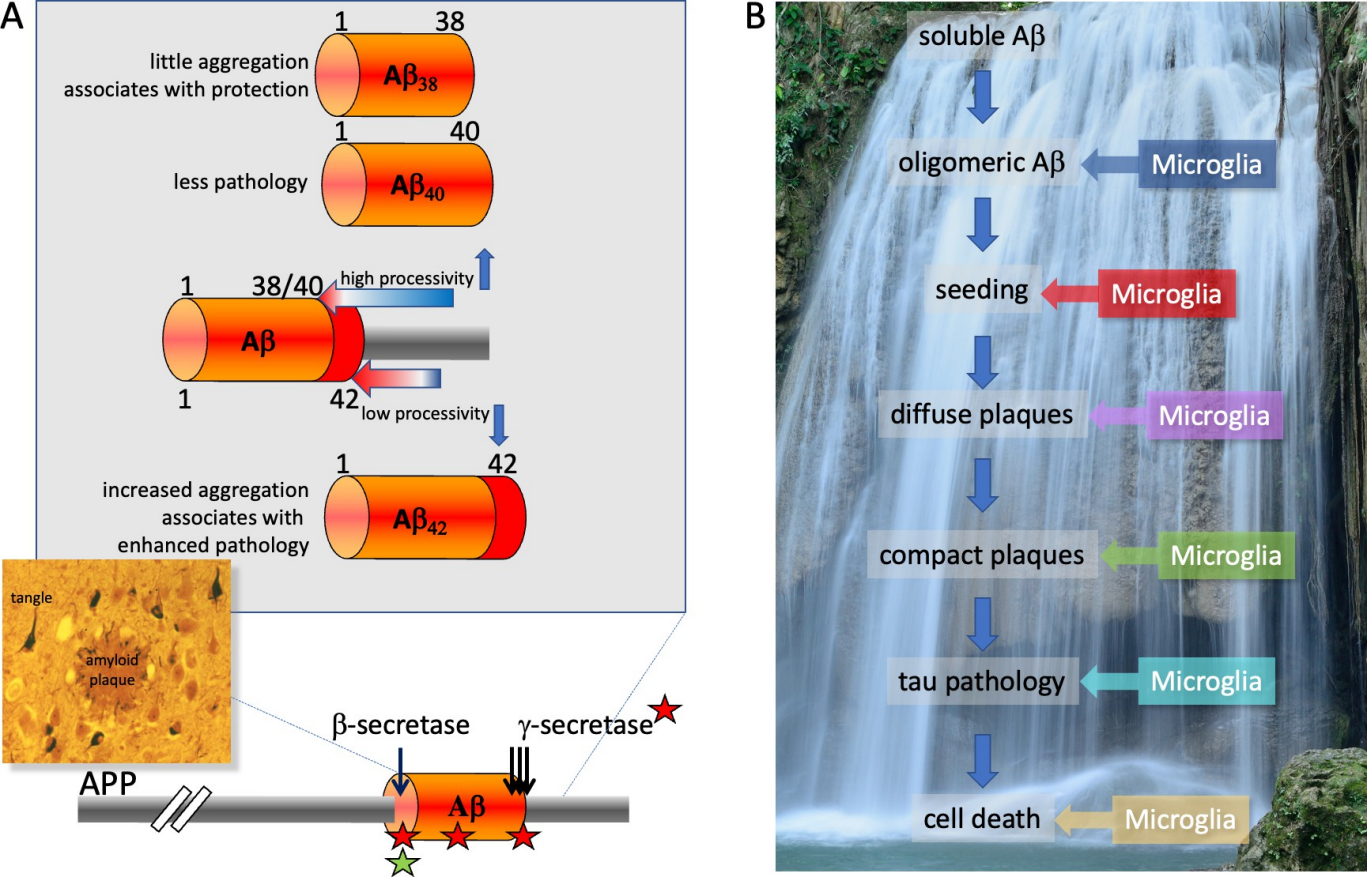
Aβ can trigger AD. (**A**) Proteolytic processing of APP by β-secretase and γ-secretase leads to the generation of Aβ protein. Red asterisks: mutations that cause familial AD; green asterisk: a protective mutation. Insert: typical amyloid plaques and neurofibrillary tangles of AD pathology. (**B**) One way to depict the amyloid cascade. Individual steps in the cascade may evoke distinct microglial responses. Aβ, amyloid β-protein; AD, Alzheimer disease; APP, amyloid precursor protein.

For decades, scientists have focused their research on a presumably stereotyped neuropathology, namely amyloid plaques and neurofibrillary tangles (Fig 1A), both of which are found in all patients with AD. Amyloid plaques are composed of abnormal aggregated forms of the amyloid β-proteins (Aβ) that are generated normally by enzymatic cleavage from the amyloid precursor protein (APP) (Fig 1A) [4–6]. Amyloid plaques are extracellular, whereas neurofibrillary tangles, composed of aggregated tau proteins, occur within neurons. How are these defining lesions connected, and what triggers the pathology initially? Based on overwhelming genetic evidence (discussed below), Aβ accumulation and its aggregation into amyloid plaques is capable of initiating the disease and is therefore often placed at the top of a theoretical cascade of events which, via multiple steps, leads to widespread neuronal dysfunction and death (Fig 1B) [6–9]. This rather linear view of molecular events has been challenged by the proposed “cellular phase” of AD which, instead of the long-pursued neurocentric view, brings the virtually simultaneous interplay of different types of brain cells, and not just neurons, into focus [10]. As a consequence, alternative pathways, some of which may be independent of Aβ accrual, might also trigger the disease. In this sense, AD may be thought of as a syndrome that has many different causes. But did we really miss the main pathogenic triggers and need to completely reorient AD research?

In this Unsolved Mystery, we argue that this rather catastrophic view is not correct. We describe a multitude of preclinical findings that strongly support the abnormal accrual of Aβ as the principal trigger of the disease. Then, we document how recent clinical trials targeting Aβ were certainly not a complete failure but rather resulted in lowering of amyloid plaque pathology in the brain and even reduced tau alteration and neurodegeneration, formally establishing disease modification. Furthermore, these anti-Aβ antibody trials slowed cognitive decline in at least some individuals, although not yet to the extent one would like to achieve.

## Is there convincing preclinical evidence for accumulation of Aβ as a key trigger of AD?

### Evidence from genetics

#### Down syndrome and AD pathology

Human genetics revolutionized AD research by identifying genetic risk factors for AD, as well as mutations in genes that definitively cause AD. In the latter cases, these genetic causes lower the conventional age of symptom onset by decades, leading to familial forms of AD. But even before the dawn of modern AD genetics, a pivotal finding pointed to the importance of Aβ as a cause of the disease. When scientists cloned *APP*, they quickly learned that the gene is located on chromosome 21 [11], a finding that was important because patients with trisomy 21 have Down syndrome and invariably develop neuropathological and clinical signs of early-onset AD, starting in their 40s or earlier. The reason for this early-onset AD could not be simpler, as the extra copy of *APP* leads to a lifelong overproduction of Aβ [12,13]. This interpretation is supported by the finding that, in rare cases of translocation Down syndrome in which only parts of chromosome 21 are duplicated, if the translocated parts contained *APP*, the patients developed AD [14], but when the duplicated part did not contain *APP*, they did not develop the disease [15]. Moreover, close study of Down syndrome enabled the discovery of temporal stages in AD pathology [13]. Young people with Down syndrome who lacked AD symptoms already had so-called diffuse plaques, largely nonfilamentous deposits of Aβ that are not associated with visible surrounding cytotoxicity. However, starting around age 20 to 30 years, these plaques slowly became more fibrillar, resembling precisely the amyloid plaques that occur in patients with AD. Thus, early on, it was apparent that a lifelong increase in Aβ production could cause AD.

#### Early-onset familial AD

**Fig 2:**
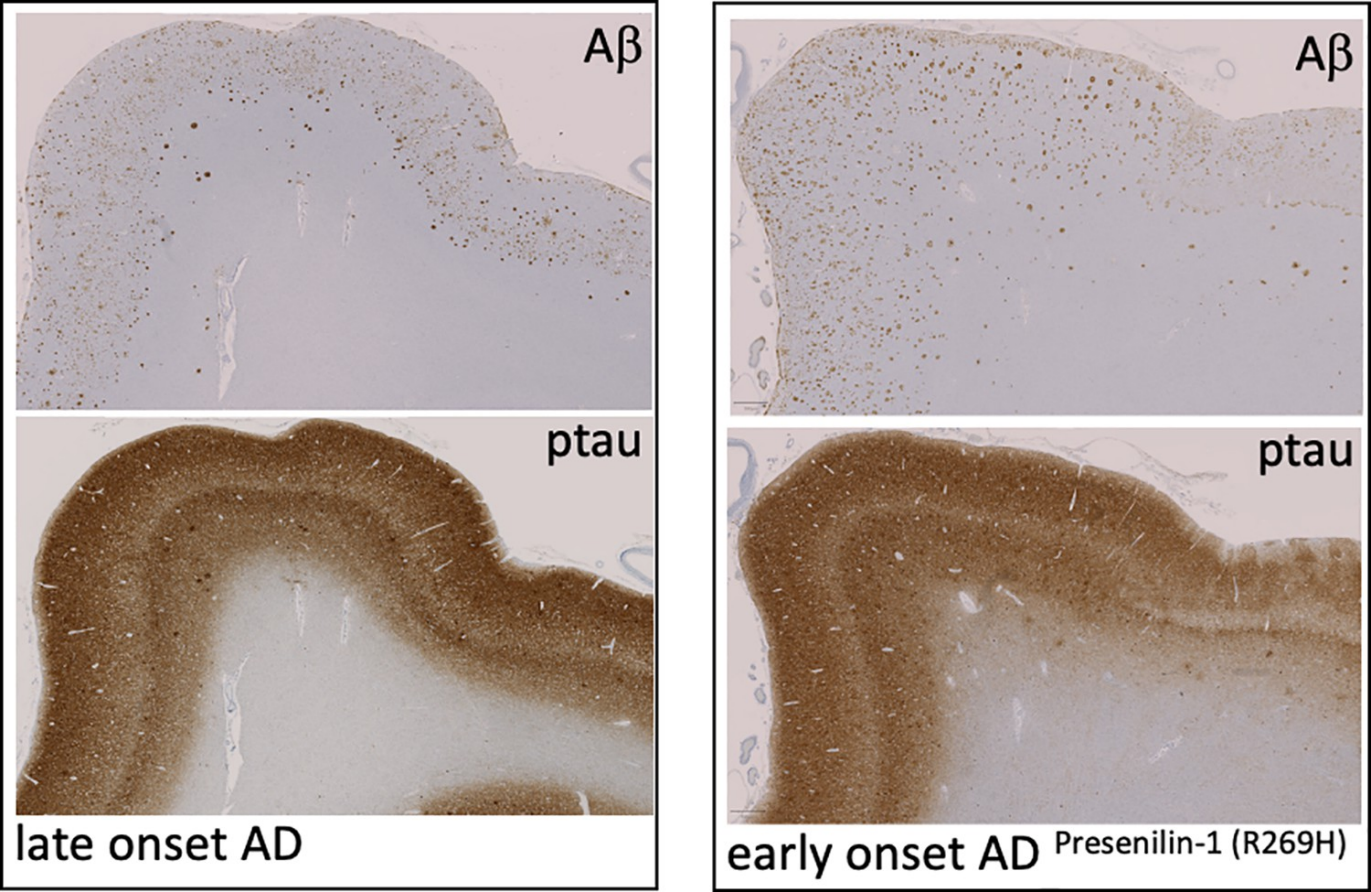
Pathology of early-onset and late-onset AD. Similar Aβ protein and tau pathology in a tissue sample from a patient with “sporadic” late-onset AD and an individual with a mutation in *PS1*, encoding presenilin-1, which causes early-onset familial AD. Shown is the gyrus parahippocampalis immunostained with the antibodies 4G8, which labels Aβ, and AT8, which labels ptau. Figure kindly provided by Dr. Thomas Arzberger (Center for Neuropathology and Prion Research, Ludwig-Maximilians-Universität, Munich). Aβ, amyloid β-protein; AD, Alzheimer disease; ptau, phospho-tau.

The movie “Still Alice” caused greater public awareness of a rare form of AD that is strictly inherited. In families with inherited AD, certain gene mutations cause a very aggressive, early-onset variant of AD which is transmitted to children with a 50% probability. Moreover, patients, who have such mutations in their genome invariably develop the disease. Although it is argued that these familial variants of AD are rare and may not be related to the “sporadic” late-onset form of AD, the amyloid plaque and tangle pathologies are practically identical (Fig 2). Not surprisingly, the pathogenic mechanisms of the genes that are mutated in familial AD represent a holy grail for deciphering AD, and a worldwide hunt for the causative genes was initiated in the early 1990s. Today, these genes have been identified, and indeed, they hold the key not only for understanding AD mechanistically, but also for the development of therapeutic strategies. Only 3 distinct genes have been identified to harbor disease-causing mutations in dominantly inherited AD [6]. The first to be recognized was *APP* itself [16,17]. One of the early *APP* variants identified contained the so-called “Swedish” mutation [18], which is located right at the cleavage site recognized by β-secretase, the enzyme that first cuts APP to initiate Aβ production (Fig 1A). Strikingly, the Swedish mutation changes the cleavage site in a way that makes it more readily cleaved by β-secretase. As a consequence, an approximately 3-fold overproduction of Aβ is observed in patients with this mutation [19,20]. Here, we have a second example, beside the triplication of *APP* in Down syndrome, where simple overproduction of Aβ is causative of AD. *APP* variants caused by mutations at the other end of the Aβ region (Fig 1A) are more complicated. Such mutations do not simply increase Aβ production but can result in the generation of longer Aβ species that are 42 or even 43 amino acids long (Aβ_42_ and Aβ_43_, respectively), rather than the more common 40-residue Aβ [21]. Such longer, more hydrophobic peptides facilitate self-aggregation of Aβ and therefore enhance the formation of neurotoxic oligomeric aggregates (see below) and the deposition of fibrillar amyloid plaques. Mutations within the central region of Aβ seem to change the structure of Aβ in a way that drives its self-aggregation [22]. Thus, AD-causing mutations in *APP* either increase total production of Aβ or generate more aggregation-prone peptides. Since peptide aggregation is a concentration dependent event [23], all AD-linked mutations in *APP* promote amyloidogenesis.

What about the much more common AD-causing mutations in the homologous presenilin-1 (PS1) and presenilin-2 (PS2) genes? Strikingly, all these mutations increase the relative levels of the longer Aβ_42_ and Aβ_43_ species, similar to the carboxyl-terminal APP mutations (Fig 1A) [24,25]. But how do these mutations affect APP processing so selectively? It turned out that *PS1* and *PS2* encode the catalytically active subunit of γ-secretase [26,27], the protease that effects APP carboxyl-terminal cleavage and thus the final liberation of Aβ. Mechanistically, it is now well described that *PS1* and *PS2* mutations slow the proteolytic performance of γ-secretase [28]. This exceptional protease mediates stepwise cleavages every 3 or 4 amino acids (termed “processivity”), starting several amino acids carboxyl-terminal to the final cleavage site (Fig 1A). The AD-causing mutations in *PS1* and *PS2* reduce this processive cleavage of APP by the PS1/PS2–γ-secretase complexes (because the mutant presenilin falls off the APP substrate too soon) and therefore increase the likelihood that longer, more hydrophobic Aβ with higher aggregation propensities are produced. Moreover, in patients with sporadic late-onset disease, higher levels of the short Aβ_38_, which arises from more efficient processivity of PS1/PS2–γ-secretase (Fig 1A), are associated with a lower degree of AD-related changes [29]. Strikingly, there are more and less aggressive *PS1* and *PS2* mutations, which now can be explained mechanistically by a correlation of greater reduction of processive APP cleavage with an earlier age of onset of symptoms [28]. Thus, early-onset familial AD is caused by mutations in the substrate for Aβ production (APP) or the proteases (presenilins) performing its release, and all these mutations enhance the aggregation propensity of Aβ and thus drive AD via increased amyloid pathology. Genetic evidence thus unequivocally positions Aβ at the top of the cascade that triggers familial AD (Fig 1B).

#### Can lifelong lowering of Aβ prevent dementia?

If increased Aβ production, as observed in patients with Down syndrome or the Swedish *APP* mutation, drives AD pathogenesis, what about reduced Aβ production? After all, the genetic evidence would strongly imply that Aβ lowering strategies may be therapeutically useful—a goal that is being actively pursued in clinical research (see below). In a large genetic project involving a substantial portion of the Icelandic population, geneticists searched for gene variants that protect against AD and age-related cognitive decline. Strikingly, a mutation within *APP* was found right at the β-secretase cleavage site that decreased Aβ production by approximately 40% throughout life [30]. Moreover, this variant not only protected from AD but also seemed to prevent age-dependent cognitive decline. Reduced Aβ deposition was confirmed in a Finnish family [31], in which the same mutation in *APP* was identified in a patient who died at 104 years of age with very little amyloid pathology. Thus, a lifelong decrease in Aβ production protects from AD. Together with the effects of the familial AD-causing mutations that increase Aβ production and aggregation, this work strongly supports a disease-triggering role of excessive Aβ. Moreover, these findings recommend Aβ as a primary target for disease modifying strategies, since nature has shown that a lifelong lowering of Aβ by approximately 40% could enable humans to live for extended periods without being affected by cognitive decline.

### Relationship with tau and other AD phenotypes

Aβ exists in many conformers (assembly states), but what are the most disease-relevant species and do they trigger tau pathology? In an analogy to fulfilling Koch’s postulates for infectious diseases, the injection of soluble Aβ oligomers (oAβ) isolated from the brains of patients with AD into healthy rodents consistently induces memory impairment and altered microglia (see below) resembling those in human AD [32,33]. Likewise, applying human oAβ to rodent or human neurons induces neuritic dystrophy and hyperphosphorylation of tau at epitopes relevant to AD [34,35]. These cellular abnormalities can be prevented by monoclonal antibodies directed at human Aβ, demonstrating the molecular specificity of the AD-like effects [34,35]. Such studies using bona fide human oAβ suggest that diffusible oligomers that are in a complex equilibrium with fibrillar Aβ in plaques are a principal pathogenic species in AD. This concept is in accord with the development of AD-like neuropathology in mice in which AD-causing mutations in human Aβ have been inserted (knocked-in) to their *APP* gene [36]. Indeed, these so-called “APP-humanized” mice accumulate oAβ and develop amyloid fibrils that are structurally like those from the brains of patients with familial AD, based on the latest cryo-electron microscopy analyses [37]. The APP knock-in mice and earlier transgenic mouse models of AD that express familial AD mutations in APP all develop not only amyloid, neuritic, astrocytic, and microglial pathologies, but also cognitive abnormalities and even biomarker evidence of neurodegeneration [38,39]. Although mice expressing mutant APP do not spontaneously develop bona fide tau pathology, a deficiency that is sometimes used to criticize the amyloid cascade hypothesis, they do show biomarker evidence of neurodegeneration, including increased phospho-tau in the brain. Moreover, a mutant APP transgenic mouse model was used to discover the amyloid-clearing effects of anti-Aβ immunotherapy, leading directly to current clinical trials [40,41].

### Role of immune cells

Due to the unequivocal preclinical evidence supporting a pivotal role of altered Aβ in triggering AD, numerous laboratories have focused their research on the mechanisms of Aβ generation and its metabolism. Moreover, since *APP* is strongly expressed in neurons, and since AD pathology is exclusively observed within the brain, AD research became rather neurocentric over the years. This narrow focus changed dramatically when geneticists identified a set of risk variants for sporadic (late-onset) AD that were principally expressed within microglia, innate immune cells of the brain, but not in neurons or other brain cells [42]. This realization caused a landslide-like shift in AD research away from neurons to microglia, and a fundamentally new topic, sometimes referred to as the cellular phase of AD, was scrutinized [10]. The cellular phase now describes a more holistic view, in which multiple cell types in addition to neurons contribute to disease onset and pathogenesis. So, the cascade may not be as linear as it was originally depicted. However, one should not forget that even Alois Alzheimer had described apparent microglial pathology [1], although he did not know the identity of these cells, and work by other pioneers in the field suggested that microglia might be invariably involved in AD pathology [43].

One of the key microglial genes identified was *TREM2* [44,45], variants of which significantly increase the risk for late-onset AD. AD risk variants in *TREM2* have surprising and unexpected functional consequences. Microglia are a very dynamic population of functionally different types of cells, ranging from a resting homeostatic population that constantly surveil the brain, to a responsive population, so-called disease-associated microglia (DAM), which actively fight brain pathology [46]. TREM2 is a receptor on the surface of microglia that senses pathological changes in the brain and is responsible for initiating the switch in gene expression pattern to allow microglia to adopt a defensive disease response [46]. Strikingly, certain loss-of-function mutations in *TREM2* prevent this switch of microglia and arrest them in a homeostatic state, even under disease conditions, whereas other mutations reduce ligand binding and signaling [47–49]. Thus, microglial activation is, at least to some extent, a protective event, and reduced activation results in a significantly enhanced risk for AD [42]. This can be directly observed in the brains of patients with AD, in which microglia are attracted to amyloid plaques, but such microglial clustering is reduced by AD risk variants in *TREM2* and almost absent upon a complete genetic loss of TREM2 in mice [50,51]. Strikingly, in humans, the TREM2-dependent protective response is triggered by deposition of amyloid seeds (small aggregates of Aβ), which drive the further aggregation and deposition of Aβ [23,52,53]; this can happen >20 years before familial AD patients show cognitive decline [54]. Moreover, amyloid seeding is clearly enhanced in the absence of functional TREM2 [50]. Thus, the earliest detectable amyloid deposition seems to drive a TREM2-dependent protective response (Fig 1B). Moreover, microglia seem to interact with all other steps of the amyloid cascade such as diffuse (nonfibrillar) and mature (largely fibrillar) plaques [50,55] and tau-triggered cell death [56] (Fig 1B). One may even speculate that there might be individual microglial populations that are specialized to deal with the different pathological challenges of AD. Thus, microglial activation is an integral component of the amyloid cascade and not just an alternate disease-causing pathway unrelated to amyloid-driven events. Finally, Aβ-triggered high levels of TREM2 have even been associated with reduced brain shrinkage and a better cognitive outcome in patients [54,57]. Major attempts are currently underway to develop TREM2-modulating therapeutic strategies. It is likely that they would be most beneficial if employed together with anti-amyloid treatments in combinatorial clinical trials [42].

Microglia may also link another key AD risk factor to amyloid-related pathology, namely apolipoprotein E (ApoE). ApoE comes in 3 variants, ApoE2, ApoE3, and ApoE4, of which ApoE4 considerably increases the risk for sporadic (late onset) AD [58]. ApoE is a component of amyloid plaques [59] and is believed to be involved in Aβ aggregation and clearance [59–61]. Blocking ApoE expression in APP transgenic mice therefore leads to disruption of plaques, which can then liberate cytotoxic oAβ (see below) and/or cause local microvascular damage by Aβ accumulating in blood vessels [62]. ApoE expression is part of the DAM response (see above), and ApoE levels are greatly increased in microglia as soon as they respond to damage [46]. In AD, this occurs in microglia clustering around amyloid plaques [50] and, since the ApoE protein is secreted, its local extracellular concentration may then increase at sites of injury. Therefore, it is conceivable that ApoE may temporarily help to keep plaques compacted to avoid liberation of oAβ that could confer local synaptotoxicity [63]. However, the ApoE4 variant is a major risk factor of AD and believed to enhance Aβ aggregation much more than the 2 other variants. Thus, it seems that very early on when plaques are seeded by oligomeric forms of Aβ, ApoE4 may efficiently facilitate Aβ deposition and therefore initiation of the disease cascade, whereas later on, when plaques have matured, microglial-derived ApoE may help to keep plaques compacted. This possibility also implies that Aβ-lowering treatment approaches are confronted with multiple and maybe even opposite consequences of Aβ accumulating in deposits. Moreover, the very early response of microglia in patients with AD and the ability of these cells to remove amyloid seeds also implies that presymptomatic treatment with amyloid-lowering drugs is likely to be most successful. In fact, aducanumab, the only currently Food and Drug Administration (FDA)-approved anti-Aβ antibody (see below), prevents oAβ seeding in a mouse model more effectively than certain other candidate anti-Aβ antibodies that do not bind to newly formed plaques [53].

Taken together, the above overwhelming preclinical evidence supports Aβ as the major pathogenic factor that triggers AD and strongly suggests that Aβ-lowering therapies should be beneficial. Then why are the clinical findings apparently so disappointing?

## Do translational and clinical studies support the preclinical evidence of Aβ pathogenicity?

### Evidence from human biomarker studies

The development of radiotracer imaging agents that can bind and visualize fibrillar amyloid plaques via positron emission tomography (PET) provided a breakthrough for studying the natural history of AD in humans [64] and later quantifying the plaque-clearing effects of anti-Aβ antibodies [65,66]. Subsequently, distinct PET ligands were invented for imaging the fibrillar deposits of tau in tangles and neurites [67]. A decade before the first PET imaging of plaques and tangles, immunochemical assays measuring their constituent proteins in cerebrospinal fluid (CSF) were developed [68], and quantifying soluble Aβ_42_ monomers and phosphorylated and total tau levels led to the development of commercial tests that can confirm a clinical diagnosis of AD. The findings that mutations in *PS1*, *PS2*, and *APP* invariably cause early-onset AD (see above) has led to the systematic measurement of several fluid and imaging biomarkers to establish an approximate sequence of the biological changes that precede symptoms [69]. This approach indicated that the earliest AD biomarker change was a gradual decline of free Aβ_42_ monomers in CSF due to their aggregation into oligomers and plaques in the brain, followed by an increase of microglial-produced soluble TREM2 [54], the detection by PET of fibrous amyloid plaques, and somewhat later, decreased cerebral metabolism (visible using fluorodeoxyglucose-PET scans) and progressive cerebral atrophy (as seen on MRI scans). Analogous biomarker and clinical profiles performed on patients with sporadic (late-onset) AD are consistent with this sequence of lesion accrual over time [70], again indicating that sporadic and familial AD may share similar mechanisms. While such biomarker-based timelines are not precise and miss some pathobiological changes (e.g., neurotoxic oAβ invisible to PET), they can help guide the timing of therapeutic interventions. The advent of tau PET scans to image tangles and dystrophic neurites has revealed that tau accumulates in the medial temporal lobe in most healthy aging humans by the time they reach their sixties, but in people developing AD, tau accumulation can spread into neocortical regions as amyloid plaques build up [71]. Indeed, there is increasing evidence of a dynamic pathogenic relationship: oAβ can bind to the outside of neurons and perturb their metabolism in a way that helps drive insolublization and tangle formation of tau inside neurons. But exactly how does this occur? We do not yet know.

### Evidence from anti-Aβ antibody clinical trials

Anti-Aβ monoclonal antibody therapy is the most advanced treatment paradigm that has been tested to date in patients with AD. Recent trial data suggest that several anti-Aβ antibodies can robustly clear amyloid plaques and produce variable slowing of declines in cognition and activities of daily living [65,66,71–73]. Thus, anti-Aβ antibodies represent the first therapeutic agents that can modify pathology in patients with AD. Of these antibodies, aducanumab [72] was the first to receive FDA approval [71] but has not yet been approved by the European Medicines Agency (EMA). Unfortunately, the 2 Phase III aducanumab trials were imperfectly executed and yielded inconsistent results that have sparked ongoing controversy. The positive 302 (EMERGE) trial showed a statistically significant 22% reduction in worsening of the Clinical Dementia Rating–Sum of Boxes (CDR-SB) test in participants who received high-dose (10 mg/kg/mo) aducanumab compared with those who received placebo (P = 0.0120) [71]. Statistically significant treatment effects were also observed for activities of daily living. This was supported by a striking dose-dependent reduction of amyloid plaques and, in a small minority of trial participants tested, a decrease in CSF phospho-tau, a marker of tangles, which correlates with cognitive decline better than plaques. This and subsequent antibody trial data (below) show that lowering Aβ can lead to less tau accumulation, strongly supporting the amyloid cascade hypothesis.

**Fig 3:**
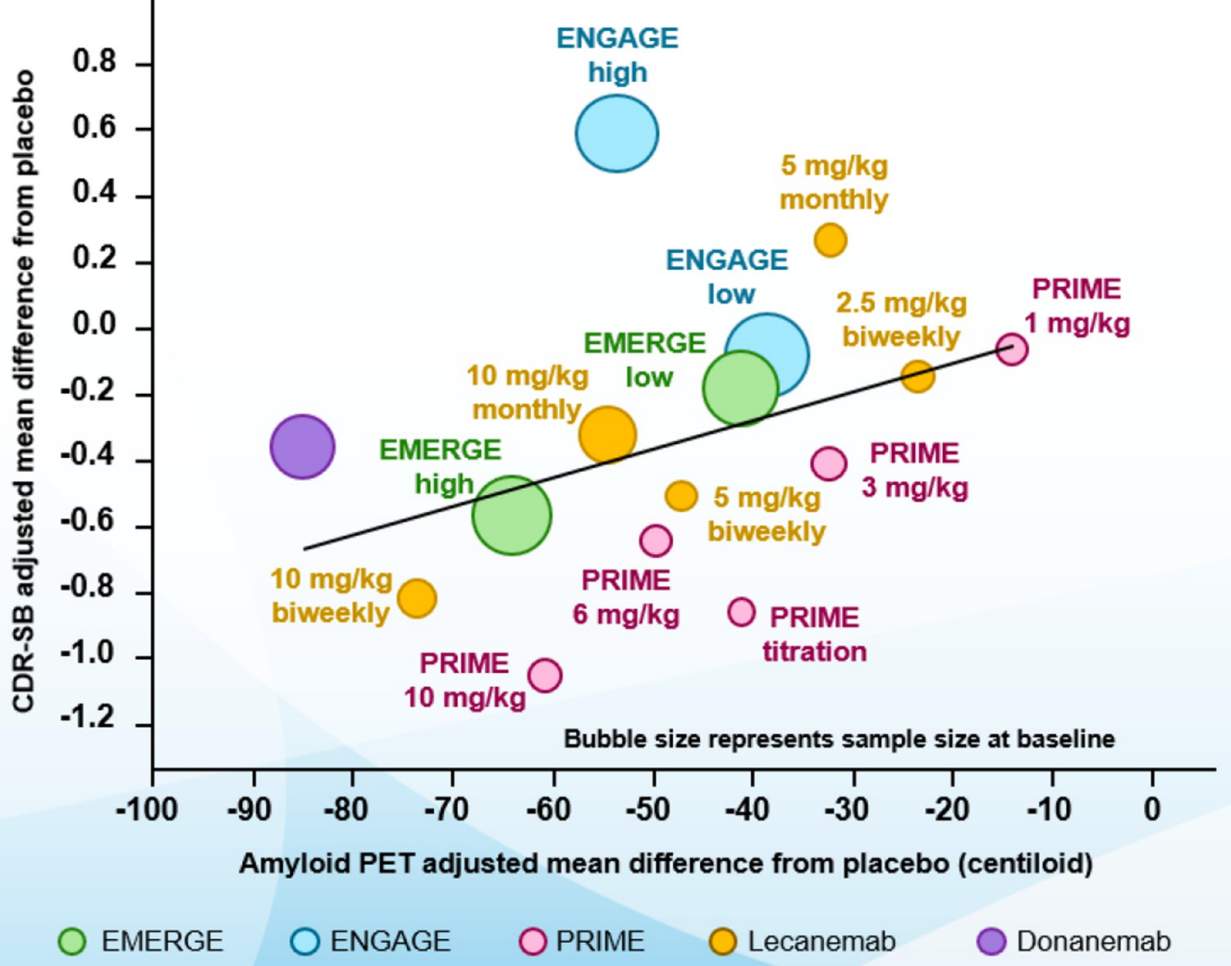
Relationship of plaque lowering and less cognitive decline across anti-Aβ antibody trials. Graphical relationship between amyloid-lowering effect (abscissa) of various anti-Aβ antibodies (“compounds”) in individual completed therapeutic trials and their effects on clinical outcome on the CDR-SB test (ordinate). The trend line moving toward the lower left corner across these trials signifies the relationship. (Obtained from reference [74]). Aβ, amyloid β-protein; CDR-SB, Clinical Dementia Rating-Sum of Boxes; PET, positron emission tomography.

In the negative 301 (ENGAGE) trial, high-dose aducanumab (10 mg/kg/mo) showed no difference in cognitive endpoints over placebo [71]. Many believed this result meant that a third clinical trial would be required before approval, but the FDA decided to use their long-standing “Accelerated Approval’ option, whereby some clinical evidence can be combined with a robust biomarker change (here, amyloid reduction) that is likely to predict future benefit. They also required Biogen (the company producing the therapy) to undertake a third clinical trial post-marketing. The FDA specifically noted “those patients [in trial 301] with higher exposure to the 10 mg/kg dose had treatment effects similar to patients in study 302 with comparable dose exposure” [74]. Furthermore, based on extensive clinical trial simulations, the FDA concluded “the probability of observing the overall positive findings … under the null assumption that aducanumab is the same as placebo was extremely low” [75]. Most importantly, the FDA stated that “there is a clear relationship between reduction of amyloid-β plaque burden in brain and preserving of clinical function in the aducanumab program, which is consistent across all 6 other available programs of anti-amyloid-β antibodies under development over the past decade. A larger reduction of amyloid-β plaque level in brain is clearly associated with better maintenance of function as measured by CDR-SB” [76] (Fig 3).

The FDA’s accelerated approval of aducanumab occurred in the context of published trial data on 2 other anti-Aβ antibodies. Lecanemab completed a large Phase II trial that narrowly missed its ambitious 12-month primary outcome, but at 18 months, it showed a favorable drug–placebo difference of 27% less decline on one cognitive test and 56% less on another [65] However, during enrolment, the EMA prohibited further ApoE4-positive patients from receiving the highest dose (10 mg/kg/2 weeks; due to fear of adverse effects). To adjust for this, the highest and next-highest dose groups (10 mg/kg/2 weeks and 10 mg/kg/mo) were analyzed together, thereby achieving balance in the number of ApoE4-positive participants with the placebo arm, and now the first cognitive test showed 20% less decline in those receiving lecanemab than in those receiving placebo (*P* = 0.053). Moreover, lecanemab reduced cognitive decline more in ApoE4-positive than in ApoE4-negative patients [65], suggesting that there could have been a greater benefit if more ApoE4-positive patients had been allowed the highest dose. The donanemab Phase II trial robustly lowered amyloid plaque levels and achieved its primary end point on a composite score of cognition and daily living activities (*P* = 0.04) [66]. Prespecified tau PET analyses suggested less tau accumulation in frontal and temporal cortex [66], again supporting a pathogenic relationship between Aβ deposition and tangle accrual. As regards the thorny question of the clinical meaningfulness of the findings across these 3 antibodies, the AD field lacks widely validated quantitative guidelines for what constitutes clinical meaningfulness from a patient and family perspective, which is all that matters.

Finally, it is often stated that many clinical trials targeting Aβ have failed, including those using inhibitors of the Aβ-generating secretases, thus seeming to negate this as a target. However, these clinical trials were conducted before the aforementioned antibody trials and did not include measurement of brain amyloid levels by PET. They included agents with safety concerns that limited dosage or caused adverse effects that halted the trials prematurely or had other pharmaceutical deficiencies. For these and other known reasons (Table 1), these earlier “failed” trials do not constitute rigorous scientific evidence against the amyloid hypothesis, despite their often being quoted as such.

**Table 1:** Apparent reasons for “failure” in some previous anti-amyloid clinical trials.

Agent	Target and mechanisms	Liabilities that precluded adequate trial effects
AN1792	Vaccine to Aβ_1–42_	Adverse effect (T-cell meningitis) halted trial after 1 to 2 doses
R-flurbiprofen	Putative γ-secretase modulator	Very poor blood–brain barrier penetration
Tramiprosate (Alzhemed)	Blocks oAß-heparan sulfate proteoglycan interactions?	No evidence of amyloid lowering
Scyllo-inositol	Compound targeting Aβ aggregation	No evidence of amyloid lowering
Semagacestat	γ-Secretase inhibitor	Therapeutic Index <3, Notch inhibition and severe adverse effects
Bapineuzumab	First anti-Aβ antibody	First observation of ARIA-E led to very low dosing
Solanezumab	Antibody to Aβ monomers	Approximately 25% of trial patients did not have AD; no plaque lowering documented
β-Secretase inhibitors	5 different compounds inhibiting β-secretase (BACE)	Adverse side effects on other BACE substrates largely halted trials

Aβ, amyloid β-protein; ARIA-E, amyloid-related imaging abnormalities-edema; BACE, β-amyloid cleaving enzyme.

## How do we move from the mixed outcomes of anti-amyloid trials to real success?

This overview of preclinical and clinical research on AD biology indicates that Aβ dyshomeostasis is an early, invariant, and necessary feature of AD pathogenesis. Then why has only one anti-amyloid agent achieved regulatory approval, and even then, under highly controversial circumstances? The most plausible explanation that emerges from available knowledge is that translating the robust preclinical and biomarker science of Aβ pathobiology into clear-cut clinical benefit has been logistically difficult and fraught with missteps. In our view, anti-amyloid trials have often included inadequate compounds, less than ideal patient selection, initiation of treatment too late in the biological process, and faulty trial execution, including premature trial termination and the expectation that slowing this chronic disease can be accomplished in just 12 to 18 months. The seemingly improved execution of the current Phase III antibody clinical trials (lecanemab, donanemab, and gantenerumab [77]) suggests that we may soon obtain more convincing evidence that sustained amyloid lowering leads to decreased pathological tau, less neurodegeneration, and the blunting of cognitive and functional decline. It would therefore be highly unwise to slow or abandon our efforts to confirm anti-Aβ therapeutic candidates, particularly since alternative, albeit highly attractive, targets (such as tau, ApoE4, and microglial modulation) are well behind Aβ lowering in the quest to lessen the disease course for patients.

If genetic, biochemical, animal modeling, fluid biomarker, and imaging studies all support Aβ as a rational target, and anti-Aβ immunotherapy reduces markers of neurodegeneration and provides some cognitive benefit, what can bring us to full success? It will be quantitative preclinical confirmation that certain antibodies (and other types of therapeutic agents) efficiently lower and neutralize oAβ as well as amyloid seeds in vivo, followed by the rigorous design and meticulous execution of clinical trials in humans confirmed to have AD pathobiology and treated for at least 18 to 24 months, with validated markers of AD pathology and multiple cognitive and functional end points that confirm each other. Of special importance would be the development of fluid and imaging markers of synaptic dysfunction, since the latter is a particularly important correlate of AD pathobiology.

Rapid progress in designing assays that sensitively quantify several key biomarkers of the AD process in blood has offered the prospect of more accessible detection of AD pathobiology and the use of plasma biomarkers as a step to select patients with AD for participation in clinical trials. The most promising markers to date are soluble fragments of tau [78] or specific phosphorylated epitopes of tau [79,80]. There is also a commercial test that quantifies Aβ_42_/Aβ_40_ ratios in plasma by mass spectrometry [81]. Ongoing research should reveal whether these and other markers of canonical AD neuropathology can be used to monitor the progression of AD and even serve as biomarker evidence of efficacy, thereby helping to validate trial outcomes. Unfortunately, no blood assays have yet been validated to monitor activation of the brain’s immune cells in AD, owing in part to the considerable separation of the peripheral immune system from that of the brain. However, certain markers such as a cleaved form of TREM2 can be measured in CSF [54,57,82]. These and other innate immune proteins may become important tools to stratify patients for trial enrollment and outcome.

It may be that only prolonged treatment in the presymptomatic stage of disease will provide a way to prevent AD, but the latest data from anti-Aβ antibody trials in mildly symptomatic patients suggest that benefit can be achieved there, too. And if intravenous antibodies prove successful, we will continue to move toward subcutaneous administration and to active vaccines that generate an endogenous polyclonal response to oAβ in a logistically feasible and economical manner. In the context of 4 decades of modern Alzheimer research, these advances may well lie just ahead.

## Abbreviations

Aβ: amyloid β-protein
AD: Alzheimer disease
APP: amyloid precursor protein
CDR-SB: Clinical Dementia Rating-Sum of Boxes
CSF: cerebrospinal fluid; DAM, disease-associated microglia
EMA: European Medicines Agency
FDA: Food and Drug Administration
oAβ: Aβ oligomer
PS1: presenilin-1
PS2: presenilin-2
PET: positron emission tomography
